# Opportunistic Pulmonary *Bordetella hinzii* Infection after Avian Exposure

**DOI:** 10.3201/eid2112.150400

**Published:** 2015-12

**Authors:** Aude Fabre, Clarisse Dupin, François Bénézit, Julien Goret, Caroline Piau, Stéphane Jouneau, Sophie Guillot, Francis Mégraud, Samer Kayal, Benoit Desrues, Alain Le Coustumier, Nicole Guiso

**Affiliations:** Centre Hospitalier de Cahors, Cahors, France (A. Fabre, A. Le Coustumier);; Centre Hospitalo-Universitaire de Bordeaux, Bordeaux, France (A. Fabre, J. Goret, F. Mégraud);; Centre Hospitalo-Universitaire de Rennes, Rennes, France (C. Dupin, F. Benezit, C. Piau, S. Jouneau, S. Kayal, B. Desrues);; Institut Pasteur, Centre National de Référence de la coqueluche et des autres bordetelloses, Paris, France (S. Guillot, N. Guiso)

**Keywords:** *Bordetella hinzii*, opportunistic pulmonary infection, avian exposure, persistence, bacteria, France, respiratory infections

## Abstract

We report 2 cases of pulmonary *Bordetella hinzii* infection in immunodeficient patients. One of these rare cases demonstrated the potential transmission of the bacteria from an avian reservoir through occupational exposure and its persistence in humans. We establish bacteriologic management of these infections and suggest therapeutic options if needed.

## Abstract 2

Diagnosing infections involving this species by routine methods is difficult.

*Bordetella hinzii* bacteria cause respiratory infections in birds and have been isolated from rodents on rare occasions (*1*,*2*). Pulmonary infection, digestive infection, and bacteriemia in humans have been reported (*3*–*5*). *B. hinzii* can persist for >1 years in the respiratory tract of humans (*5*), but its transmission from birds has not been proven. Through 2 new cases and a brief review of the literature, we show a possible association between occupational risk and pulmonary colonization by *B. hinzii*. Then, we suggest how to manage these infections in humans, although pathogenicity of this bacterium remains unclear.

## Clinical Cases

### Case-Patient 1

In April 2013, a 43-year-old man was admitted to the Pneumology Service of the University Hospital Centre of Rennes (Rennes, France) because of fatigue, fever, and exacerbation of bronchiectasis. He had undergone an allograft bone marrow transplantation and therapy with corticosteroids and chemotherapy for an acute myeloid leukemia diagnosed 15 months earlier but was in remission during the infectious episode. After the leukemia was diagnosed, he stopped working; his occupation had involved cleaning pipes and waste tanks with high-pressure water in poultry abattoirs.

The patient was a former smoker with a medical history of type 1 diabetes, vascular hypertension, and nonsymptomatic chronic bronchiectasis before the allograft. He was hospitalized for 2 episodes of pulmonary infections in October 2012 and February–March 2013, during which *Escherichia coli* was isolated and for which he received ciprofloxacin.

On admission, he was febrile (39°C) despite chemoprophylaxis with trimethoprim/sulfamethoxazole, posaconazole, and valaciclovir. The patient had dyspnea and dry cough without sputum production. Physical examination found crackles at bases of both lungs. Thoracic radiograph showed a cardiomegaly, an interstitial syndrome, and pleural effusion.

Laboratory analyses showed anemia (hemoglobin 92 g/L [reference range 130–170 g/L]) and hyperleukcocytosis (12.4 G/L [reference range 4–10 G/L]) with 8.3 G/L (reference range 2–7.5 G/L) polynuclear neutrophils (PNN). The inflammatory syndrome was confirmed by the elevated C-reactive protein concentration (44.7 mg/L [reference range <5 mg/L]). Calculated antimicrobial therapy was started on the second day after admission with piperacillin/tazobactam and ciprofloxacin.

Bacteriologic cultures of sputum collected on admission yielded 10^9^ CFU/mL *B. hinzii*. Bronchial aspiration and bronchoalveolar lavage fluid were collected for microbial investigations 2 days later and showed 10^7^ CFU/mL *B. hinzii* and 3 × 10^5^ CFU/mL *Staphylococcus epidermidis*, respectively. Polymicrobial flora were present in all cultures performed, indicating that those samples were contaminated by oropharyngeal flora.

Laboratory analyses for nocardiosis, pneumocystosis, aspergillosis, and tuberculosis were negative. Multiplex PCR for herpes simplex virus, varicella zoster virus, cytomegalovirus, and Epstein-Barr virus and culture for influenza viruses A and B, human herpesvirus 6, adenovirus, metapneumovirus, and parainfluenza viruses 1–3 did not detect any of these viruses.

Because of the persistence of symptoms, the patient’s antimicrobial therapy was changed, following a decision after 5 days to target *Staphylococcus*, to parenteral vancomycin for 10 days. Clinical improvement of pulmonary signs and symptoms was observed after 15 days. In addition, physical rehabilitation was initiated to support the malnutrition and muscle atrophy. The patient returned home after 1.5 months of rehabilitation.

### Case-Patient 2

In September 2014, a 74-year-old man hospitalized in a private clinic for partial laryngectomy and a tracheotomy because of a second recurrence of laryngeal cancer was transferred to the intensive care unit of the University Hospital Center of Bordeaux (Bordeaux, France) for decompensation of chronic obstructive pulmonary disease 11 days later. His medical history included vascular hypertension, dyslipidemia, prostate cancer in 2007 that required total surgical excision, and ischemic heart disease in 2011. He also had chronic obstructive pulmonary disease that had not been documented or treated and a laryngeal cancer for which he underwent a cordectomy in 2002 and a partial laryngectomy in 2007. The patient was a former airline pilot living in the city; he had not had any pets for many years and had had only rare contact with poultry during childhood.

On admission, he had fever (38.7°C), respiratory distress with hypoxemia, and purulent tracheal secretions discharging from the tracheotomy orifice. Physical examination found a high heart rate (107 bpm). Pulmonary auscultation was normal. Thoracic radiograph showed systematic alveolar images on the right. Results of laboratory tests revealed anemia (88 g/L [reference range 130–170 g/L]) and an inflammatory syndrome with hyperleucocytosis (11.8 G/L [reference range 4–10 G/L]) with 11.3 G/L (reference range 2–7.5 G/L) PNN.

Tracheal aspiration was performed on admission and bronchoalveolar lavage 6 days later and samples were cultured. Tracheal secretions yielded 10^6^ CFU/mL *B. hinzii* and 6 × 10^7^ CFU/mL methicillin-resistant *S. aureus*. Bronchoalveolar lavage fluid gave 9 × 10^2^ CFU/mL *B. hinzii* and 10^2^ CFU/mL methicillin-resistant *S. aureus*. *B. hinzii* was also cultured on ESBL medium (bioMérieux, Marcy l’Etoile, France) from a rectal swab sample taken for systematic research for the carriage of resistant bacteria.

Antimicrobial therapy was started on admission to intensive care with piperacillin/tazobactam (7 days) and vancomycin (11 days). The patient was transferred after 14 days, following clinical pulmonary recovery, to the private clinic for otorhinolaryngeal care.

## Microbiological Investigations

Direct microscopic examination of specimens showed substantial presence of PNNs and gram-negative short bacilli, except in the bronchoalveolar lavage fluid from case-patient 2, in which no bacteria was observed. In both case-patients, colonies were apparent after 24 or 48 hours of incubation (37°C, 5% CO_2_) on PolyViteX chocolate agar. In case-patient 2, they also grew on trypcase soy agar with 5% horse blood and Haemophilus chocolate agar with bacitracin (bioMérieux). The colonies were medium sized (1–2 mm), smooth, round, convex, and grayish; those from case-patient 2 were very mucoid. Microscopic examination of colonies showed gram-negative short bacilli. Identification was inconclusive with Vitek 2 system IdGN cards (bioMérieux) and RapID NH (Remel, Lenexa, KS, USA). The Api 20NE strip (bioMérieux) identified *B. avium* based on the score 0000067 with high percentage (96.7%) and typicality (T = 1). Routine identification by matrix-assisted laser desorption/ionization time-of-flight (MALDI-TOF) mass spectrometry (Brucker Daltonik GmbH, Germany) gave *B. hinzii* with good scores (T = 2.29 for case-patient 1 and T = 2.35 for case-patient 2). Final identification for both cases involved amplification and sequencing of 1,480 nt of 16S rRNA gene, then use of the blastn program (http://www.ncbi.nlm.nih.gov/BLAST/) and the GenBank database. The nucleotide sequences showed the best BLAST hit with *B. hinzii* (99.9% homology in both cases). Antimicrobial susceptibility testing was performed on Mueller-Hinton medium by an Etest method (bioMérieux) and MICs determined (Table).

**Table:** Antimicrobial drug susceptibility testing for human *Bordetella hinzii* isolates by the Etest method*

Antimicrobial drug	Case-patient 1†	Case-patient 2†	BC-306 (*11*)	BC-305 (*11*)	BL-3210 (*3*)	Hristov et al. isolate 1 (*10*)‡	Hristov et al. isolate 2 (*10*)‡	Hristov et al. isolate 3 (*10*)‡
Amoxicillin	ND	32	16§	16§	12§	ND	ND	ND
Amoxicillin/clavulanic acid	ND	ND	32	16	ND	ND	ND	ND
Ampicillin/sulbactam	ND	ND	16/8	16/8	ND	ND	ND	ND
Piperacillin	0.38	ND	ND	ND	ND	32	ND	ND
Piperacillin/tazobactam	0.25	0.75	1	1	ND	ND	ND	ND
Ticarcillin	ND	ND	ND	ND	ND	>32	>64	ND
Ticarcillin/clavulanic acid	ND	32	32	64	ND	ND	ND	ND
Cefoxitine	ND	>256	ND	ND	ND	ND	ND	ND
Cefotaxime	ND	>32	ND	ND	>32	ND	ND	ND
Ceftriaxone	ND	ND	64	64	ND	ND	ND	>32
Ceftazidime	1.5	2	4	4	4	ND	ND	ND
Cefepime	ND	6	8	8	ND	>16	>16	>16
Ertapenem	3	0.023	ND	ND	ND	ND	ND	ND
Imipenem	0.75	1.5	2	2	1	ND	ND	ND
Meropenem	1	0.125	ND	ND	ND	≤1	8	4
Doripenem	0.5	0.125	ND	ND	ND	ND	ND	ND
Aztreonam	ND	ND	ND	ND	ND	>16	>16	ND
Gentamicin	0.75	4	2	4	4	≤1	4	≤2
Tobramycin	4	48	ND	ND	ND	>8	>8	4
Amikacin	2	16	ND	ND	ND	16	≤4	≤8
Clarithromycin	4	ND	ND	ND	ND	ND	ND	ND
Clindamycin	>256	ND	ND	ND	ND	ND	ND	ND
Rifampin	1	>32	N	ND	ND	ND	ND	ND
Levofloxacin	>32	0.25	2	2	ND	ND	ND	ND
Ciprofloxacin	>32	0.75	4	4	>32	>2	>2	>2
Moxifloxacin	4	0.5	ND	ND	ND	ND	ND	ND
Cotrimoxazole	0.38	ND	0.047	0.023	ND	≤0.5/9.5	2/38	ND
Vancomycin	>32	>256	ND	ND	ND	ND	ND	ND
Teicoplanin	>32	>256	ND	ND	ND	ND	ND	ND
Daptomycine	>256	>256	ND	ND	ND	ND	ND	ND
Linezolid	>256	>256	ND	ND	ND	ND	ND	ND
Fusidic acid	>32	ND	ND	ND	ND	ND	ND	ND
Tetracycline	ND	ND	ND	ND	0.38	ND	ND	ND
Minocycline	1	ND	ND	ND	ND	≤2	>8	ND
Tigecycline	0.5	0.38	ND	ND	ND	ND	ND	ND
Colistin	0.38	0.094	ND	ND	ND	ND	ND	ND
Fosfomycine	>1024	>1024	ND	ND	ND	ND	ND	ND

*Values are MICs as determined by Etest in μg/mL ND, not determined.  †This study. ‡Isolates 1, 2, and 3 of Hristov et al. case correspond to respiratory isolates (1 and 2) and blood isolate (isolate 3). §Ampicillin rather than amoxicillin was tested.

## Discussion

*B. hinzii* is found in the respiratory tract of poultry. Although it was initially described as a commensal bacterium in birds, some veterinary isolates appear to have pathogenic properties: Register et al. reported that it is associated with tracheal lesions in turkeys (*1*,*6*). *B. hinzii* has been sporadically described in rabbits (*6*) and rodents (*2*) and had been reported to cause pulmonary disease in laboratory mice (*7*) and bacteremia in rats (*2*). 

Few cases have been described in humans. Most involved bacteremia or respiratory or digestive infections (*3*–*5*). *B. hinzii* was first isolated in 1957 from the sputum of a patient in France but was misidentified as *Alcaligenes faecalis* (*8*). In 1994, Cookson et al. described *B. hinzii* bacteremia in an AIDS patient (*4*). Two other cases of bacteremia in immunodeficient patients were associated with the isolation of *B. hinzii* from the pulmonary tract (*9*,*10*).

Two digestive infections have been reported. A fatal case was described in an immunocompetent man (*11*) with cholestasis and bacteremia. The second case was a biliary infection in a liver transplant recipient: *B. hinzii* was isolated from 4 bile samples during a 6-month period (*3*), demonstrating that colonization by *B. hinzii* can be long-lasting.

*B. hinzii* appears as an opportunistic pathogen causing respiratory infections in cystic fibrosis patients (*5*,*12*). Other respiratory infections in both immunodeficient and immunocompetent persons have been reported. Gadea et al. isolated *B. hinzii* associated with *Nocardia asteroides* in a bronchoalveolar lavage sample from an AIDS patient (*13*). Palacian Ruiz et al. described a *B. hinzii* respiratory infection in an immunocompetent elderly woman that was associated with *Klebsiella oxytoca* (*14*). *B. hinzii* was isolated 8 times during a 1-year period from the respiratory tract of a cystic fibrosis patient (*5*).

*B. hinzii* infections are presumably underdiagnosed because of misidentification by the routine phenotypic identification procedures that are the basis for Phoenix (Phoenix BD Diagnostic Systems, Sparks, MD, USA) and Vitek (bioMérieux) automated systems and for the API 20NE strip manual system (bioMérieux). Indeed, *B. hinzii* is not referenced in the API 20NE database and often gives the same score as *B. avium*. The API 20NE database should be updated and the score 0000067 should be referred to as *B. avium*–*B. hinzii* complex. The mean score for 6 clinical isolates analyzed with the MALDI-TOF mass spectrometry system (Brucker Daltonik GmbH) by the French National Reference Centre of pertussis and other bordetelloses was T = 2.31, with T = 2.063 as the minimal score. MALDI-TOF is useful to discriminate *B. hinzii* from other *Bordetella* species (*15*). Sequencing the 16S rRNA gene is the most reliable technique to confirm the species, but the MALDI-TOF system is more suitable for routine identification and would enable more cases to be detected.

Although poultry seems to be the major reservoir (*1*), we cannot exclude the possibility that mammals such as rabbits and rodents also could be potential reservoirs (*2*,*7*). Humans can become infected by aerosols from the avian reservoir, which probably was the route for case-patient 1, who had a pulmonary infection long after exposure. Survival in the digestive tract is another specificity of *B. hinzii* among the *Bordetella* species. This bacterial survival was illustrated for case-patient 2, who had a positive culture from a rectal swab sample; the bacteria most likely was transmitted by the oral route, possibly after ingestion of contaminated poultry products (*3*) or deglutition of respiratory secretions.

Several reports have demonstrated the prolonged persistence of *B. hinzii* in the respiratory and digestive tracts (*3*,*5*); such persistence, at least in the respiratory tract, is also observed for *B. petrii* and *B. bronchiseptica* (*16*). This persistence may explain why infection can develop long after exposure and makes identification of the source difficult.

The characteristics of all the reported cases suggest that *B. hinzii* is an opportunistic pathogen in humans. Other pathogens were isolated in several cases such that the extent of its pathogenicity remains obscure. Surprisingly, *Staphylococcus* sp. was the most frequently associated pathogen, raising the possibility of synergy between these 2 bacteria (*5*). The role of *B. hinzii* is still unclear in immunocompetent patients, where it may act as colonizer. For case-patient 2, the mucoid aspect of the colonies is in accordance with prolonged carriage, as described for *Pseudomonas aeruginosa* (*17*).

Treatment of nonclassical *Bordetella* infections is not standardized. The interpretation of antimicrobial sensitivity testing is not established and is usually done by inference from other nonfermentative gram-negative rods. According to in vitro sensitivity testing for human isolates described in the literature and pharmacology, piperacillin/tazobactam and carbapenems (excluding ertapenem) may be effective (Table). Optimal duration of treatment has not been established but should be long enough to cure the infection and, if possible, eliminate the bacteria. Both patients reported here were initially treated with piperacillin/tazobactam without clinical improvement despite the sensitivity of the isolates to this association; presumably, the treatment duration was too short. The curative doses needed to eliminate *B. hinzii* appear to be high because *B. hinzii* was isolated from case-patient 1 despite prophylactic treatment with trimethoprim/sulfamethoxazole, a combination to which the isolate was susceptible. Numerous discrepancies exist between the results of disk diffusion and MIC tests (*5*), as observed for *B. bronchiseptica* (A. Ce Coustumier, unpub. data). MIC testing should be performed to confirm the sensitivity of any such isolates. We can speculate about possible antimicrobial resistance acquisition, regarding fluoroquinolone resistance in case-patient 1 and his exposure to this pharmacologic class during previous hospitalizations, as described elsewhere (*3*), but data remain insufficient to prove it. Microbiological tests after the episode are required to evaluate the effectiveness of treatment.

In conclusion, we report here 2 cases of *B. hinzii* pulmonary infection in immunodeficient patients, probably after avian exposure. Although the transmission could not be clearly established, a potential link exists between the occupational exposure and the isolation of *B. hinzii* in the pulmonary tract. The respiratory or digestive carriage can be prolonged such that the infection might emerge only long after contamination, making identification of the source difficult. Although *B. hinzii* is well established as opportunistic, microbiologists and clinicians need to be aware of the difficulty in diagnosing infections by this species using routine methods. Identification of all clinical isolates belonging to nonclassical *Bordetella* sp. should be confirmed by a reference laboratory. Further clinical and microbial investigations are necessary to understand the epidemiology and the pathogenicity of *B. hinzii*. Optimal antimicrobial treatments need to be established and supported by pharmacology and antimicrobial in vitro sensitivity testing (bacteriostasis, bactericidy).
